# Long term outcome and clinical experience on immune tolerance induction therapies in infantile Pompe disease

**DOI:** 10.1186/1471-2474-14-S2-O8

**Published:** 2013-05-29

**Authors:** Stephanie Austin, Priya Kishnani

**Affiliations:** 1 Department of Pediatrics, Duke University Medical Center, Durham, North Carolina, USA

## 

Pompe disease (glycogen storage disease type II) is an autosomal recessive lysosomal storage disorder caused by a deficiency of the enzyme acid alpha-glucosidase. Enzyme replacement therapy (ERT) with alglucosidase alfa has resulted in a clinical benefit in a subset of patients. Cross-reactive Immunological Material (CRIM)-negative status is associated with poor prognosis. Patients with CRIM-negative infantile Pompe disease mount a strong immune response against alglucosidase alfa ERT, resulting in a clinical decline and death despite therapy. Most develop high sustained antibody titers. Based on our clinical and laboratory experience, about 40 percent of patients with infantile Pompe disease develop HSAT. Early identification of patients at risk is needed to allow for treatment intervention with immune tolerance induction (ITI) protocols. These protocols have included the use of agents such as rituximab, methotrexate, IVIG, and agents that target the plasma cells. Different treatment approaches are needed for patients with Pompe disease treated with ITI in the naïve setting as compared to patients with high sustained antibody titers. Without ITI, the CRIM-negative patients do poorly on ERT alone. We will present a successful global experience in 15 cases and discuss the safety, efficacy and feasibility of a clinical algorithm developed at our institution to identify CRIM-negative status and allow timely initiation of ERT and ITI in this vulnerable population. Our data show that the clinical algorithm of rapidly diagnosing and initiating ITI coincident with the start of ERT in CRIM-negative patients is feasible and can be achieved in a timely manner.

